# Human Genetics of Diabetic Retinopathy: Current Perspectives

**DOI:** 10.1155/2010/172593

**Published:** 2010-07-13

**Authors:** Daniel P. K. Ng

**Affiliations:** Department of Epidemiology and Public Health, Yong Loo Lin School of Medicine, National University of Singapore, 16 Medical Drive MD3, Singapore 117597

## Abstract

Diabetic retinopathy (DR) is a most severe microvascular complication which, if left unchecked, can be sight-threatening. With the global prevalence of diabetes being relentlessly projected to rise to 438 million subjects by 2030, DR will undoubtedly pose a major public health concern. Efforts to unravel the human genetics of DR have been undertaken using the candidate gene and linkage approaches, while GWAS efforts are still lacking. Aside from evidence for a few genes including aldose reductase and vascular endothelial growth factor, the genetics of DR remain poorly elucidated. Nevertheless, the promise of impactful scientific discoveries may be realized if concerted and collaborative efforts are mounted to identify the genes for DR. Harnessing new genetic technologies and resources such as the upcoming 1000 Genomes Project will help advance this field of research, and potentially lead to a rich harvest of insights into the biological mechanisms underlying this debilitating complication.

## 1. Introduction

Diabetic retinopathy (DR) is a most severe microvascular complication which if left unchecked can be sight-threatening. DR ranks as a common cause of blindness worldwide, particularly among adults [1–3]. With the global prevalence of diabetes being projected to rise to 438 million subjects by 2030, DR will certainly pose a major public health concern [4]. 

The presence of diabetic retinopathy is evidenced by the appearance of retinal microvascular lesions. Early changes include microaneurysms, hemorrhages, hard exudates, cotton wool spots, intraretinal microvascular abnormalities, and venous beading and characterize nonproliferative diabetic retinopathy (NPDR). The more severe state of proliferative diabetic retinopathy (PDR) is marked by the formation of abnormal fragile new blood vessels that are prone to hemorrhage. Finally, visual impairment results in secondary to pre-retinal or vitreous hemorrhage and diabetic maculopathy.

## 2. Familial Clustering of DR

Epidemiological studies have shown that the prevalence of DR increases with diabetes duration and various clinical measures, primarily intensive glycaemic control, can delay the development of DR [5, 6]. It is however noteworthy that some patients may still develop DR even with good glycaemic control. Conversely, some patients with poor glycaemic control are spared from this complication and notably, in long surviving patients with type 1 diabetes, the association between diabetic retinopathy and glycaemic control is less well supported [7]. Genetic susceptibility may underlie this observation, a proposal that was supported by twin analysis conducted more than three decades ago [8]. Of late, this early evidence for a role of genetic factors in DR has been corroborated by familial aggregation studies among patients with either type 1 or type 2 diabetes (Table 1). Familial clustering also extends across different ethnicities. This effect of genes likely influences the various stages of DR including NPDR, PDR, and macular edema although different genes may impact specific stages of disease [9–13].

## 3. Candidate Genes for DR

The search for DR genes has predominantly been undertaken using the candidate gene approach. The case-control study design is generally employed and is appropriate for detecting both major and minor genes. The candidate gene approach requires a fair knowledge of the pathogenic mechanisms underlying DR and this has benefitted from the many years of research in this field [14–16]. Several pathways and processes have been strongly implicated including the renin-angiotensin system, polyol pathway, nonenzymatic glycation, endothelial dysfunction, vascular tone maintenance, extracellular matrix remodeling, and angiogenesis which is dysregulated in diabetes leading to proliferation of new fragile retinal capillaries that culminate in PDR [17, 18]. Correspondingly, a host of genes involved in these pathways/processes have been treated as potential candidate genes. These genes include angiotensin-I converting enzyme (*ACE*), angiotensin II type 1 receptor (*AGTR1*), angiotensinogen (*AGT*), vascular endothelial growth factor (*VEGF*), aldose reductase (*AR2*), receptor for advanced glycation endproducts (*RAGE*), glucose transporter 1 (*GLUT1*), inducible and constitutive nitric oxide synthases (*NOS2A*, *NOS3*), transforming growth factor beta (*TGFbeta*), endothelin isoforms, and its cellular receptors, among others [19–28].

As is the current thought in the field of complex genetics, the effect sizes of these genetic factors are likely to be modest although major genes have been postulated to exist. Consequently, individual studies have, more often than not, yielded inconsistent and even conflicting findings [29]. To circumvent this issue, meta-analyses have been undertaken to pinpoint the few genes for which there might be cumulative evidence for an association with DR. Three of these genes are highlighted in the following sections.

## 4. Aldose Reductase (AKR1B1, Human Chromosome 7q35)

Aldose reductase (AKR1B1) is the rate-limiting enzyme of the polyol pathway, which catalyzes NADPH-dependent reduction of glucose to sorbitol. AKR1B1 has been reported in human pericytes, and activation of this pathway has been strongly implicated in the pathogenesis of DR. Notably, retinal vascular changes such as microaneurysm formation and degeneration of retinal pericytes may be induced in rats and dogs that have been made hyperglycemic by a diet rich in galactose, the latter being reduced by AKR1B1 to form galactitol [14, 15]. The search for pharmacological inhibitors of this enzyme for use in the treatment of DR is ongoing [30]. In a recent meta-analysis of the various polymorphisms in *AKR1B1*, the Z-2 allele of the (CA)_n_ microsatellite located at the 5′ end of the gene showed the most significant association with diabetic retinopathy (OR = 2.33, 95% CI = 1.49–3.64, *P* = .0002), independently of the type of diabetes present. This association was present regardless of whether cases had NPDR (OR = 1.64, 95% CI = 1.14–2.35, *P* = .0075) or PDR (OR = 1.51, 95% CI = 1.16–1.97, *P* = .0023) [31]. Conversely, the Z + 2 and Z alleles conferred protection against DR [31]. Beside the (CA)_n_ microsatellite, the association of the promoter SNP rs759853 and DR has also been reported in a number of studies. Meta-analysis suggested that the T allele conferred protection against DR in type 1 diabetes (OR = 0.49, 95% CI 0.36–0.68, *P* < .0001) while there was no statistically significant association in patients with type 2 diabetes [31].

## 5. Vascular Endothelial Growth Factor (VEGF, Human Chromosome 6p12)

VEGF is an important growth factor involved in causing vascular permeability. High vitreous levels have been repeatedly detected in eyes of patients undergoing vitrectomy operations for PDR and diabetic macular edema [32–35]. The cellular effects of VEGF are mediated primarily through two closely related receptor tyrosine kinases VEGFR-1 (Flt1) and VEGFR-2 (KDR/Flk1) [36]. Regulation of target genes such as heptocyte growth factor (HGF), urinary and tissue plasminogen activator (uPA, tPA), matrix metalloproteinase-9 (MMP9) is then achieved through complex signaling pathways, including through protein kinase C (PKC) [37]. VEGF inhibition has been shown to ameliorate retinal changes including retinal neovascularization and breakdown of the blood-retinal barrier [38–40]. A total of six polymorphisms (rs25648, rs1570360, rs3095039, rs35569394, rs699947 and rs2010963) in *VEGF* have been examined and of these, the G allele of rs2010963 was significantly associated with a reduced risk of NPDR in patients with type 2 diabetes (OR = 0.62, 95% CI = 0.48–0.81, *P* = .0005) [31]. Considering that VEGF has been implicated in neovascularization, it might appear surprising that none of the polymorphisms so far including rs2010963 has been significantly associated with PDR [31].

## 6. Angiotensin-I Converting Enzyme (ACE, Human Chromosome 17q23)

Among the DR candidate genes, ACE is the most widely studied. The well-known insertion deletion (I/D) polymorphism in ACE which results from the insertion/deletion of a 287 bp Alu sequence in intron 16 accounts for half the variance of serum enzyme levels. Individuals who are homozygous for the insertion allele (II genotype) have significantly lower levels of ACE compared to carriers of the deletion allele (ID and DD genotypes) [41]. A meta-analysis of six studies on this polymorphism in patients with type 1 diabetes and seven studies in patients with type 2 diabetes suggested that there was no statistically significant association of this polymorphism and the development of any form of diabetic retinopathy [31]. A second recent independent meta-analysis corroborated this finding but suggested that ACE I/D may be associated with PDR (OR = 1.37, 95% CI = 1.02–1.84) under a dominant genetic model assuming either fixed or random effects [42].

## 7. Deficiency of Candidate Gene Studies

Apart from the few genes mentioned above, the overall evidence for the remaining candidate genes investigated to date is weak [31]. Several factors are likely responsible for this situation, a primary one being small sample sizes. Some have as little as 50 subjects while larger studies with more than 100 cases are decidedly uncommon [18, 29]. Meta-analyses have been undertaken to present the overall evidence for an association but this technique has well-known drawbacks, including the possibility of publication bias which has to be carefully assessed.

Another limitation is that most studies failed to take into account the role of haplotype diversity at the candidate gene locus. Studies so far have focused on single (and often random) SNPs and, as such, cannot reasonably exclude a gene as being important in DR since linkage disequilibrium between these SNPs and the true functional SNP may be low [20–23, 26]. To circumvent this problem, it will be potentially useful to examine haplotype-disease associations. The usefulness of this approach was demonstrated recently in the case of ACE where risk haplotypes for diabetic nephropathy (another important microvascular complication of diabetes) were identified in independent studies [43, 44]. Another point of note is that the reported studies have rarely taken into account the potential influence of covariates. One such covariate is diabetes duration, the importance of which has been demonstrated through simulation studies [45] as well as in epidemiological studies seeking to identify genes for another microvascular complication of diabetes (i.e., diabetic nephropathy) [46, 47].

## 8. Linkage Studies

Besides the candidate gene approach, whole genome linkage studies have also been undertaken to identify chromosomal regions which potentially harbor major genes for DR. Three such studies have been reported and these have been conducted on Mexican Americans and Pima Indians with type 2 diabetes [48–50] (Table 2). With the possible exception of human chromosome 1p36, the linkage evidence for the other regions has not been replicated and this is undoubtedly related to the very modest LOD scores initially reported (Table 2). Genetic linkage appeared to exist for both advanced stages as well as earlier manifestations of DR [49]. The identities of the purported major susceptibility genes in these linkage regions continue to remain elusive.

## 9. Genomewide Association Studies (GWASs)

Recent advances have made it possible to genotype the human genome at up to a million polymorphic sites in thousands of samples within a reasonable time frame. This has heralded the era of genomewide association studies (GWASs) which have been successful at pinpointing a number of novel genes related to a spectrum of diseases [51]. Unfortunately, no GWAS effort to identify DR genes has been reported to date. Extrapolating from the collective GWAS experience [51, 52], one can nevertheless anticipate that for any DR gene found through GWAS, the implicated risk alleles will have limited effect sizes with OR <1.4 while the risk allele frequencies will be quite frequent in the population (>0.20). Thus, the risk alleles will be low penetrant since many individuals will harbor the risk alleles, but among these, the majority will tend to remain disease-free. In addition, GWAS will not be expected to unmask the identity of the major susceptibility genes for DR even in regions that show linkage to this disease [52]. Finally, the genes identified through GWAS will only account for a very limited proportion of the familial clustering observed for DR. Clinically, the genetic information gleaned from GWAS will have limited utility as potential disease classifiers [53]. 

However, balancing these modest expectations of GWAS lies the prospect of identifying novel genes which can undoubtedly shed fresh insight into the pathogenic pathways responsible for DR. Indeed, it is realistic to hope that elucidation of these pathways can in the long run lead to new molecular targets for pharmacological intervention. Already, newer therapies based on known pathogenic pathways particularly intravitreal antiangiogenesis agents that act to suppress VEGF are already being evaluated, although the evidence base currently does not yet support their routine use in the clinic [54]. In the interim, good glycaemic and blood pressure control necessarily remains the cornerstone in the prevention of DR [54].

## 10. Conclusion

From the current survey of the human genetics of DR, it is clear that this field remains poorly developed. However, therein lies the promise of impactful scientific discoveries if concerted and collaborative efforts are mounted to identify the genes responsible for DR. Just as the International Human Genome and HapMap Projects have propelled genetic discoveries in the past few years, the added resources of the upcoming 1000 Genomes Project (http://www.1000genomes.org/) as well as new genetic technologies may likewise prove beneficial to this field of research and lead to a rich harvest of insights into the biological mechanisms underlying this debilitating complication.

## Figures and Tables

**Table 1:** Familial clustering of DR.

Type of Diabetes	Patients/Study	Evidence for familiar Clustering	Reference
Type 1	DCCT subjects	OR = 3.1, 95% CI = 1.2–7.8	[9]
Type 1	FinnDiane Study	OR = 2.76, 95% CI = 1.25–6.11, Heritability = 52%	[13]
Type 2	Asian Indians	OR = 3.37, 95% CI = 1.56–7.29	[10]
Type 2	Mexican Americans	OR = 1.72, 95% CI = 1.03–2.88	[11]
Type 2	Find-Eye Study	Heritability = 27%	[12]

**Table 2:** Potential chromosomal regions linked to DR (LOD scores >1).

Chromosome	Definition of DR	Population	LOD score	Nearest genetic markers	Reference
1p36	Retinopathy score in worst eye	Pima Indians	3.1	D1S3669	[50]

1p36	Any DR	Mexican Americans	1.24	GGAT2A07	[49]

2q37	Severe NPDR/PDR	Mexican Americans	1.11	AFM112yd4	[49]

3p26	Severe NPDR/PDR	Mexican Americans	1.29	GATA22G12	[49]

3q12	Severe NPDR/PDR	Mexican Americans	1.40	GATA68D03	[49]

3q12	Any DR	Mexican Americans	2.41	GATA68D03	[49]

3q26	Presence of at least one microaneruysm, hemorrahage or proliferative DR	Pima Indians	1.36	D3S3053, D3S2427	[48]

7p15	Any DR	Mexican Americans	1.02	GATA41G07	[49]

9q21	Presence of at least one microaneruysm, hemorrhage or proliferative DR	Pima Indians	1.46	D9S1120, D9S910	[48]

12p13	Any DR	Mexican Americans	2.47	GATA49D12	[49]

12q23	Severe NPDR/PDR	Mexican Americans	1.03	GATA85A04	[49]

15q25	Any DR	Mexican Americans	1.07	ATA28G05	[49]

15q26	Any DR	Mexican Americans	1.16	GATA22F01	[49]
